# Menopausal hormone therapy and the incidence of carpal tunnel syndrome in postmenopausal women: Findings from the Women’s Health Initiative

**DOI:** 10.1371/journal.pone.0207509

**Published:** 2018-12-04

**Authors:** Tala Al-Rousan, Jeffrey A. Sparks, Mary Pettinger, Rowan Chlebowski, JoAnn E. Manson, Andrew M. Kauntiz, Robert Wallace

**Affiliations:** 1 Department of Global Health, Harvard T.H. Chan School of Public Health, Harvard University, Boston, Massachusetts, United States of America; 2 Division of Rheumatology, Immunology and Allergy, Brigham and Women's Hospital and Harvard Medical School, Boston, Massachusetts, United States of America; 3 Public Health Sciences Division, Fred Hutchinson Cancer Research Center, Seattle, Washington, United States of America; 4 Department of Medical Oncology and Therapeutics Research, City of Hope National Medical Center, Duarte, California, United States of America; 5 Division of Preventive Medicine, Brigham and Women's Hospital, Harvard Medical School, Boston, Massachusetts, United States of America; 6 Department of Obstetrics and Gynecology, University of Florida College of Medicine, Jacksonville, Florida, United States of America; 7 Department of Epidemiology, College of Public Health, University of Iowa, Iowa City, Iowa, United States of America; Public Library of Science, UNITED KINGDOM

## Abstract

**Importance:**

Carpal tunnel syndrome (CTS) is a common and debilitating condition that commonly affects postmenopausal women.

**Objective:**

To determine the effect of menopausal hormone therapy (HT) in healthy postmenopausal women on CTS risk.

**Design:**

We conducted a secondary analysis of the Women’s Health Initiative’s (WHI) HT trials linked to Medicare claims data. Separate intention-to-treat analyses were performed for the two trials; the conjugated equine estrogens alone (CEE alone) and the trial of CEE plus medroxyprogesterone acetate (MPA) trial. (ClinicalTrials.gov, NCT **number**): NCT00000611.

**Setting:**

Two randomized, double-blind, placebo-controlled trials conducted at 40 US clinical centers.

**Participants:**

The sample size included in the analysis was 16,053 community-dwelling women aged ≥65 years at study entry or those who later aged into Medicare eligibility, and who were enrolled in Medicare (including Part A and/or Part B coverage).

**Intervention:**

Women with hysterectomy were randomized to 0.625 mg/d of conjugated equine estrogens (CEE) or placebo (n = 8376). Women without hysterectomy were randomized to estrogen plus progestin (E+P), given as CEE plus 2.5 mg/d of medroxyprogesterone acetate (n = 14203).

**Main outcome(s):**

The primary outcome was incident CTS and the secondary outcome was therapeutic CTS procedure occurring during the intervention phases of the trials.

**Results:**

A total of 16,053 women were randomized in both trials. During mean follow up of 4.5 ± 2.8 years in the CEE trial (n = 6,833), there were 203 incident CTS cases in the intervention and 262 incident CTS cases in the placebo group (HR, 0.78; 95% CI, 0.65–0.94; P = 0.009). The CEE+MPA trial (n = 9,220) followed participants for a mean of 3.7 ± 2.3 years. There were 173 incident CTS cases in the intervention compared to 203 cases in the placebo group (HR, 0.80, 95% CI, 0.65–0.97; P = 0.027).

**Conclusions:**

These findings suggest a protective effect of menopausal HT on the incidence of CTS among postmenopausal women. A potential therapeutic role for other forms of estrogen therapy in the management of CTS warrants future research.

## Introduction

Carpal tunnel syndrome (CTS) is the most common compressive mono-neuropathy and represents an important cause of functional hand impairment and disability. CTS is often associated with loss of work, inability to perform family and social roles, living and working with pain, and physical disability that may lead to compensation claims.[1, 2] Surgical and other treatments are often needed. The cumulative estimated income loss from CTS per patient over a period of six years is estimated to be $45,000–89,000.[3] CTS therefore confers an economic burden on patients, employers, and health care institutions.[4, 5]

The estimated annual incidence of CTS ranges from 324 to 542 per 100,000 adult women, with a female to male ratio of 3 to 1.[6–8] The epidemiology of CTS varies by geography but incidence increases with age, suggesting that menopausal factors may play an important role in the pathogenesis of CTS.[9, 10] Recent evidence also suggests that CTS incidence is rising among postmenopausal women, possibly associated with declines in ovarian production of estrogen and the universal declining use of Hormone Therapy (HT).[11] It is believed that CTS occurs due to idiopathic increase in the pressure within the carpal tunnel, but may be precipitated by factors including anatomic compression from repeated use, ischemic injury, and systemic inflammation from conditions such as pregnancy and rheumatoid arthritis as well as local inflammation of the flexor tenosynovium.[12, 13]

Small case series have suggested that severe CTS unresponsive to traditional non-surgical treatments may benefit from HT prescribed for menopausal symptoms.[14, 15] However, such reports pertain to symptoms among patients already diagnosed with CTS in the context of other primary diagnoses. Furthermore, a few studies provided conflicting evidence and suggested that HT had no effect on CTS; these studies were limited by case-control methodology, short follow-up time, small sample sizes, or having arthralgia as the primary outcome rather than CTS.[16–18]

Because the association between HT and CTS remains uncertain we used the largest clinical trial of women receiving HT to date, the Women’s Health Initiative (WHI) HT trials, and followed a large sample of women aged 65 and older who were randomized in a double-blinded, placebo-controlled trial to determine the effect of HT on the incidence of CTS.

## Methods

### Participants

We conducted a secondary analysis of the estrogen-alone (Conjugated Equine Estrogens [CEE]) and CEE plus medroxyprogesterone (CEE+MPA) trials conducted within WHI. A detailed discussion of the methods of the WHI trials and the principal findings of the HT trials are available elsewhere.[19–21] The current study population consisted of women aged 65 years and older at study entry or those who later aged into Medicare eligibility, and who were enrolled in Medicare (including Part A and/or Part B coverage). WHI participants were linked with Medicare data using social security number, date of birth and, in some cases, date of death, and residential zip code.

Women were recruited at 40 clinical centers between 1993 and 1998. The National Institutes of Health and institutional review boards for all participating clinical sites approved the WHI protocols and consent forms. The National Institutes of Health Institutional Review Boards (NIH IRBs) for all participating clinical sites approved the WHI protocols and consent forms. This is a clinical trial that was approved and reviewed by the ethical reviews of the National Heart, Lung, and Blood Institute and local IRBs of 40 participating clinical sites. More information can be found at: https://clinicaltrials.gov/ct2/show/NCT00000611. Women were excluded if their expected survival was less than 3 years, had a contraindication to using the study medications, or were deemed to be at risk of poor medication adherence. Women with hysterectomy were eligible for the CEE trial, and women without hysterectomy were eligible for the CEE+MPA trial due to increased risk of endometrial cancer with unopposed estrogen. Eligible participants were randomized in equal proportions to receive 0.625 mg/d of CEE (Premarin, Wyeth, Philadelphia, PA) or placebo using a stratified permuted block algorithm with blinded dispensing.[20] Women in the CEE+MPA trial were randomized in equal proportions to receive 0.625 mg/d of CEE plus 2.5 mg/d of MPA or placebo, administered as a single tablet.

### Outcome measures

The primary outcome for this analysis was incident CTS, and the secondary outcome was CTS associated procedures. We identified outcomes in the CMS Medicare data by using a single International Classification of Diseases (9th Revision, Clinical Modification [ICD-9-CM]) code as a principal or secondary diagnosis code. We defined CTS in the CMS Medicare data as first occurrence of the ICD-9-CM diagnosis code of 354.0 in any position; in either the inpatient (MedPAR), outpatient header or carrier header data files. A CTS procedure was defined as the first occurrence of one of the three Current Procedural Terminology (CPT) codes: 1) Code: 20526 (injections, therapeutic (e.g., local anesthetic, corticosteroid), carpal tunnel, 2) Code: 29848 (endoscopy, wrist, surgical, with release of transverse carpal ligament); or 3) Code: 64721 (neuroplasty and/or transposition; median nerve at carpal tunnel) in either the outpatient or carrier line item data files. All of the other medical conditions used in the analysis were self-reported.

### Statistical analyses

An intention-to-treat analysis was used based solely on Medicare-determined outcomes among women 65 years of age or older at randomization or who aged into Medicare during the intervention period. For these analyses, we allowed participants to age into Medicare during the intervention period, so that time zero for the time-to-event models starts when each participant’s Medicare coverage started, rather than at randomization. As a result, duration of follow-up is shorter than what was previously reported for all participants in the two trials.[21] Cox proportional hazards models were used to determine the effect of treatment assignment on incidence of CTS outcomes. Baseline characteristics for women who were linked with Medicare data were compared by randomization group using Chi-square and t-tests.

We compared the incidence of CTS among the women randomized to HT or placebo during the period of active intervention. The start of follow-up was the start of Medicare coverage through July 7, 2002 for the CEE+MPA trial, and through February 29, 2004 for the CEE trial, the dates that the intervention phases of the trial stopped. Participants were censored when they were no longer enrolled in fee-for-service Medicare Part A or B, withdrew from the trial, or died. Annual rates of clinical events were estimated for the intervention period. Cumulative hazard curves estimated using the Kaplan-Meier method[22]compared study drug assignment from the start of Medicare coverage through the end of the intervention phases of the trials. HRs were estimated using Cox proportional hazards models stratified by randomization age strata (50–54, 55–59, 60–69, 70–79) and Dietary Modification Trial randomization arm, since a small proportion of HT trial participants were also randomized into that study. Since all potential confounders were balanced by randomization groups, other factors were not included in the final models. The proportional hazards assumption was tested by including an interaction term in the models between the treatment arm and the log time-to-event. The proportional hazards assumption was not met in the CEE trial models; additional models were run to test for differences in risk between two time periods of follow-up where the risk appeared to differ. Tests for interaction between potential risk factors and study medications were conducted in expanded Cox models by adding product terms between the study medication and each risk factor that included: linear age, linear trend for BMI, and categorical factors for job type, smoking, prior HT use, diabetes, rheumatoid arthritis and thyroid disease.

Sensitivity analyses for nonadherence with study medication were conducted by censoring follow-up time 6 months after participants became non-adherent, defined as either taking less than 80% of study medication or starting HT outside of the protocol. In addition, to control for characteristics that may relate to adherence, an inverse probability weighting method was used where the weights are the inverse of the participant’s estimated probability of adherence.[23]

All statistical analyses were conducted using SAS software Version 9.4 and R software Version 3.1.2

## Results

**Fig 1:**
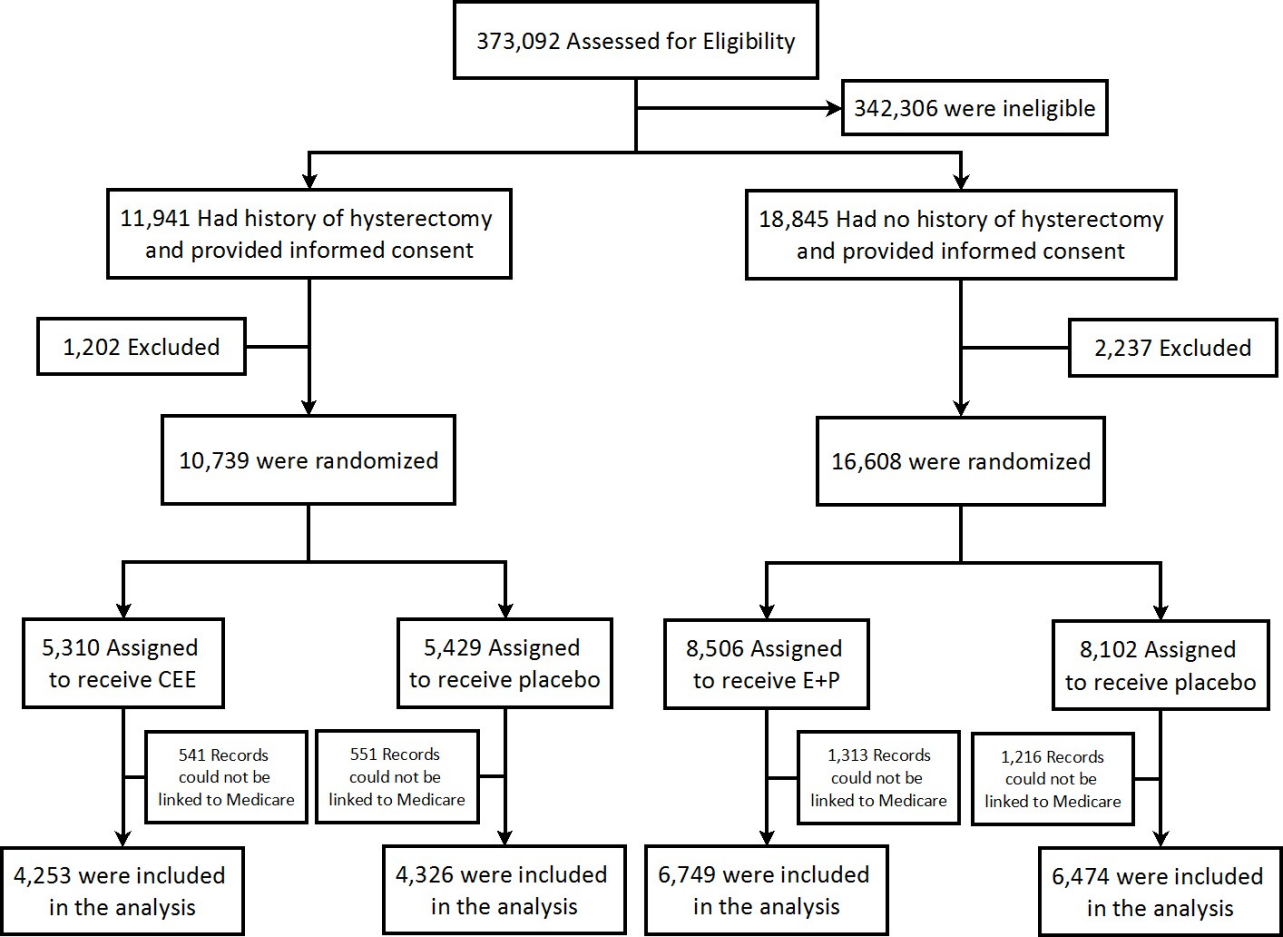
Flowchart of the woman study enrollment. Individuals were excluded prior to randomization for safety reasons such as history of breast cancer (4.5%), or clinic staff impression that a woman was not a good candidate for the study (4.8%). Some women were not included in the trial if the stratum was closed (4.6%). The majority of exclusions were due to lack of interest or no informed consent for the estrogen therapy component of the Women’s Heath Initiative (81.2%). CEE, conjugated equine estrogens; MPA, medroxyprogesterone acetate.

The study flow diagram for this analysis is outlined in Fig 1. Baseline characteristics for the entire trial cohort were presented elsewhere.[19, 24] A total of 16,053 women either had CMS coverage at randomization or later aged into it during the intervention phase and were included in the analysis. Of those women, 6,833 were in the CEE trial and 9,220 were in the CEE+MPA trial. Baseline characteristics for the study group are summarized in Table 1. The randomization groups were balanced on key baseline demographic and disease risk factors. The mean intervention intervals were 4.5±2.8 years for the CEE trial, and 3.7±2.3 years in the CEE+MPA trial.

**Table 1:** Baseline Characteristics of the women’s health initiative hormone trial participants meeting the criteria for carpal tunnel syndrome (CTS) study at start of medicare coverage (N = 16,053).

Characteristics, n (%)	Conjugated Equine Estrogens Trial		Estrogen and Progestin Trial	
	CEE (N = 3,380)	Placebo (N = 3,453)	CEE+MPA (N = 4,700)	Placebo (4,520)
Age at screening, years				
Mean (SD)	65.6 (5.9)	65.7 (6.0)	66.1 (5.4)	66.3 (5.4)
50–54	53 (1.6)	55 (1.6)	29 (0.6)	29 (0.6)
55–59	488 (14.4)	508 (14.7)	424 (9.0)	380 (8.4)
60–64	1000 (29.6)	1007 (29.2)	1548 (32.9)	1438 (31.8)
65–69	899 (26.6)	931 (27.0)	1413 (30.1)	1405 (31.1)
70–74	685 (20.3)	663 (19.2)	901 (19.2)	894 (19.8)
75–79	255 (7.5)	289 (8.4)	385 (8.2)	374 (8.3)
Race/ethnicity				
White	2660 (78.7)	2687 (77.8)	4133 (87.9)	3962 (87.7)
Black	459 (13.6)	500 (14.5)	271 (5.8)	282 (6.2)
Hispanic	150 (4.4)	159 (4.6)	147 (3.1)	137 (3.0)
American Indian	26 (0.8)	24 (0.7)	11 (0.2)	12 (0.3)
Asian/Pacific Islander	51 (1.5)	40 (1.2)	82 (1.7)	68 (1.5)
Unknown	34 (1.0)	43 (1.2)	56 (1.2)	59 (1.3)
Family Income				
< $10,000	281 (8.8)	311 (9.5)	246 (5.5)	215 (5.0)
$10,000 - $19,999	653 (20.5)	632 (19.4)	751 (16.8)	667 (15.6)
$20,000 - $34,999	1005 (31.5)	1009 (30.9)	1307 (29.3)	1294 (30.2)
$35,000 - $49,999	604 (18.9)	638 (19.5)	958 (21.5)	898 (21.0)
$50,000 - $74,999	413 (12.9)	435 (13.3)	707 (15.8)	727 (17.0)
≥$75,000	237 (7.4)	240 (7.4)	494 (11.1)	483 (11.3)
Education				
0–8 years	109 (3.3)	77 (2.2)	105 (2.2)	74 (1.6)
Some high school	232 (6.9)	261 (7.6)	199 (4.2)	211 (4.7)
High school diploma/GED	825 (24.6)	794 (23.1)	977 (20.8)	966 (21.5)
School after high school	1423 (42.5)	1460 (42.4)	1850 (39.5)	1680 (37.3)
College degree or higher	760 (22.7)	849 (24.7)	1555 (33.2)	1570 (34.9)
Body-mass index, kg/m^2^				
Mean (SD)	29.9 (5.9)	29.8 (6.0)	28.4 (5.7)	28.3 (5.7)
<25	728 (21.6)	712 (20.7)	1396 (29.8)	1394 (31.1)
25-<30	1170 (34.8)	1246 (36.3)	1680 (35.9)	1628 (36.3)
≥30	1465 (43.6)	1478 (43.0)	1607 (34.3)	1467 (32.7)
Alcohol intake				
Non-drinker	473 (14.1)	493 (14.4)	586 (12.6)	573 (12.8)
Past drinker	859 (25.7)	808 (23.6)	805 (17.3)	745 (16.7)
<1 drink per month	455 (13.6)	455 (13.3)	603 (12.9)	615 (13.8)
<1 drink per week	623 (18.6)	656 (19.2)	938 (20.1)	823 (18.4)
1 - <7 drinks per week	640 (19.1)	695 (20.3)	1143 (24.5)	1134 (25.4)
≥7 drinks per week	293 (8.8)	316 (9.2)	590 (12.6)	582 (13.0)
Smoking status				
Never	1773 (51.8)	1772 (51.9)	2332 (50.2)	2260 (50.7)
Past	1292 (38.6)	1307 (38.3)	1898 (40.9)	1808 (40.6)
Current	322 (9.6)	332 (9.7)	415 (8.9)	389 (8.7)
Type of job, current or past				
Managerial/professional	945 (32.2)	951 (32.2)	1473 (36.8)	1523 (37.6)
Technical/sales/admin	923 (31.4)	931 (31.5)	1214 (30.3)	1233 (30.5)
Service/labor	737 (25.1)	735 (24.9)	837 (20.9)	824 (20.4)
Homemaker only	333 (11.3)	335 (11.4)	484 (12.1)	469 (11.6)
Prior menopausal hormone use				
Never	1775 (52.5)	1790 (51.9)	3577 (76.2)	3434 (76.0)
Past	1234 (36.5)	1299 (37.6)	925 (19.7)	905 (20.0)
Current	371 (11.0)	362 (10.5)	195 (4.2)	179 (4.0)
Duration of prior menopausal hormone use (years)				
Never used	1775 (52.5)	1790 (51.9)	3577 (76.2)	3434 (76.0)
<5	855 (25.3)	886 (25.7)	764 (16.3)	761 (16.8)
5-<10	294 (8.7)	305 (8.8)	208 (4.4)	171 (3.8)
≥10	456 (13.5)	472 (13.7)	151 (3.2)	153 (3.4)
Medical History				
Myocardial infarction	112 (3.3)	125 (3.6)	93 (2.0)	110 (2.4)
Stroke	56 (1.7)	68 (2.0)	28 (0.6)	54 (1.2)
Diabetes	345 (10.2)	357 (10.3)	275 (5.9)	281 (6.2)
Thyroid disease	839 (25.1)	767 (22.4)	1008 (21.6)	944 (21.1)
Arthritis (rheumatoid or other)	1858 (55.5)	1918 (56.3)	2187 (47.0)	2143 (47.9)
Rheumatoid arthritis	228 (7.2)	207 (6.5)	212 (4.8)	192 (4.4)
Lupus	11 (0.3)	7 (0.2)	14 (0.3)	22 (0.5)
Osteoporosis	244 (7.3)	238 (7.0)	279 (6.0)	298 (6.7)
Bisphosphonate use	36 (1.1)	45 (1.3)	70 (1.5)	86 (1.9)
Any fracture at age 55+	519 (18.1)	483 (17.0)	734 (18.8)	737 (18.7)
Lower arm or wrist fracture at age 55+	129 (4.7)	130 (4.8)	223 (6.0)	204 (5.5)
Waist/hip ratio, mean (SD)	0.83 (0.08)	0.83 (0.08)	0.82 (0.08)	0.82 (0.08)

The observed annual incidence rate of any CTS event in the CEE trial was 1.39% for the intervention group compared to 1.76% for the placebo group (Table 2). The observed annual incidence rate of CTS was 1.01% for the CEE+MPA group, compared with 1.26% for the corresponding placebo group. Both HT groups demonstrated a significantly decreased risk of CTS (HR, 0.78; 95% CI, 0.65–0.94; P = 0.009 for the CEE trial and HR, 0.80, 95% CI, 0.65–0.97; P = 0.027 for the CEE+MPA trial).

**Table 2:** Incidence of carpal tunnel syndrome (CTS) diagnosis during hormone trial intervention periods: Sample in center for medicare & medicaid services (CMS) at randomization or later aged in during intervention (N = 16,053).

	Conjugated Equine Estrogen Trial (N = 6,833)			Estrogen & Progestin Trial (N = 9,220)		
	CEE (N = 3,380)	Placebo (N = 3,453)	p-value^1^	CEE+MPA (N = 4,700)	Placebo (N = 4,520)	p-value^1^
**CTS diagnosis**						
**Events, N (%)**	**203 (6.0)**	**262 (7.6)**		**173 (3.7)**	**203 (4.5)**	
**Annualized incidence (%)**	**1.39**	**1.76**		**1.01**	**1.26**	
**HR1 (95% CI)**	**0.78 (0.65–0.94)**	**1.00 (Ref)**	**0.009**	**0.80 (0.65–0.97)**	**1.00 (Ref)**	**0.027**
**CTS procedure**						
**Events, N (%)**	**52 (1.5)**	**69 (2.0)**		**39 (0.8)**	**46 (1.0)**	
**Annualized incidence (%)**	**0.34**	**0.45**		**0.22**	**0.28**	
**HR1 (95% CI)**	**0.77 (0.54–1.11)**	**1.00 (Ref)**	**0.164**	**0.78 (0.51–1.20)**	**1.00 (Ref)**	**0.256**

Follow-up time, mean (SD) for CEE trial = 4.5 (2.8) years and for CEE+MPA trial 3.7 (2.3) years.

^1^The Cox proportional hazards regression model stratified by randomization age strata (50–54, 55–59, 60–69, 70–79) and Dietary Modification Trial randomization arm.

**Fig 2:**
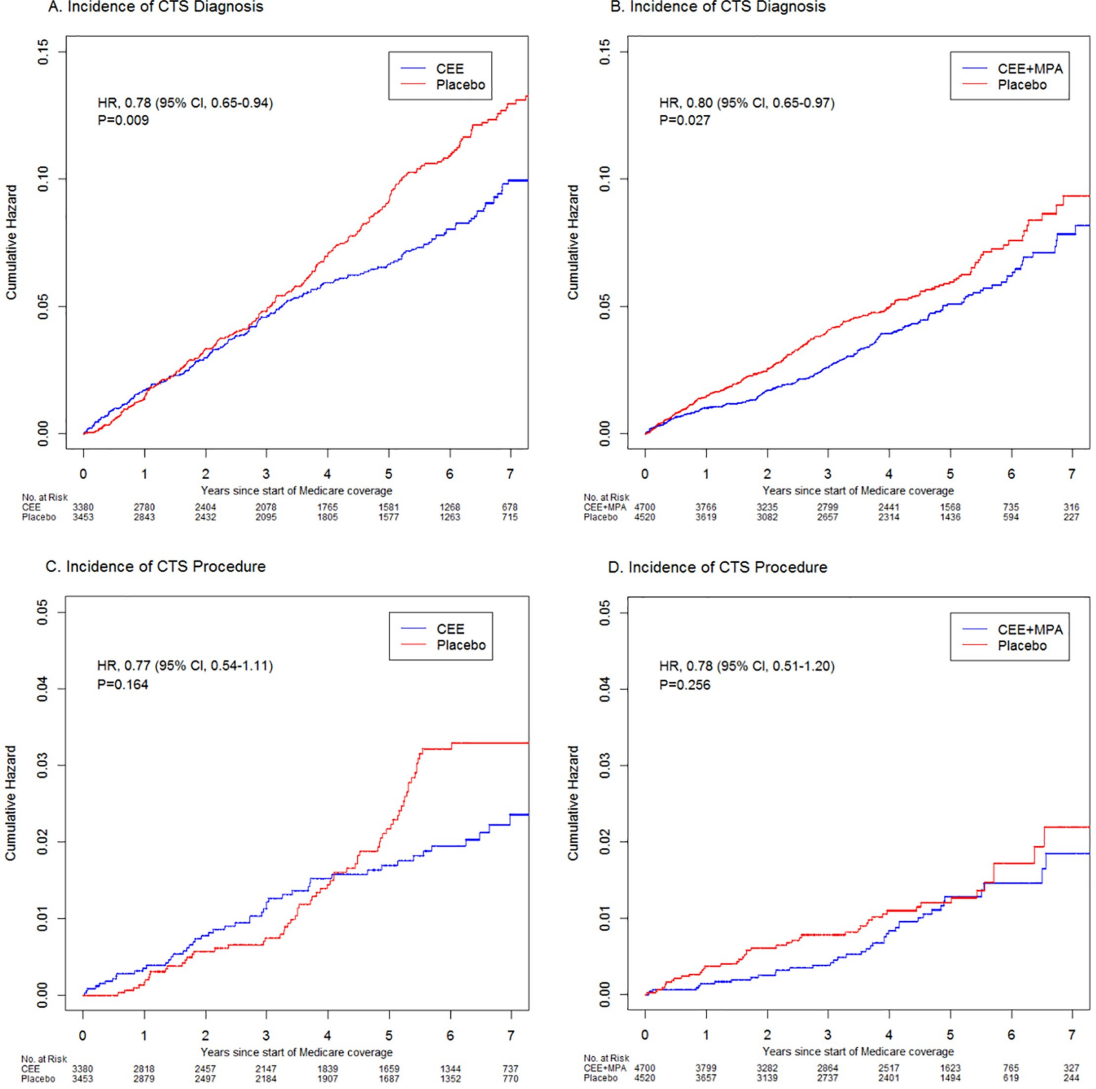
Cumulative hazard curves for carpal tunnel syndrome outcomes in the women’s health initiative (whi) estrogen alone and the estrogen and progestin trials during the intervention periods^1^. Cumulative hazard curves for CTS diagnosis in the Women’s Health Initiative CEE (panel A) and the CEE+MPA (panel B) trials during the intervention periods. Comparable plots are shown for CTS procedure in the CEE (panel C) and the CEE+MPA (panel D). The intervention reduces the cumulative hazard of CTS cases by 22% in the CEE trial and by 20% in the CEE+MPA trial. A similar trend is seen for CTS procedures but it was not statistically significant. Cox proportional hazard models were used where time zero started when each participant’s Medicare coverage started. CEE, conjugated equine estrogens; MPA, medroxyprogesterone acetate; CI, confidence interval; HR, hazard ratio. ^1^Hazard ratios (HR) and 95% confidence intervals (CI) estimated from Cox proportional hazards models; p-values based on Wald Chi-square statistics.

The cumulative incidence curves for CTS diagnosis or procedure showed lower cumulative hazard in the intervention group beginning in the first year of randomization for the CEE+MPA trial. In the CEE trial, lower cumulative hazard of CTS became apparent after approximately four years, Fig 2.

Most CTS procedures were neuroplasty and/or transposition of the median nerve at the carpal tunnel (approximately 90% of CTS procedures). When accounting for the full follow up period, there were no statistically significant differences in therapeutic procedures in either the CEE trial (HR, 0.77; 95% CI, 0.54, 1.11; P = 0.16), or in the CEE+MPA trial (HR, 0.78; 95% CI, 0.51, 1.20; P = 0.26). This is likely explained by a relatively low number of CTS procedures since point estimates were similar to the CTS incidence rates. Furthermore, in the CEE trial, there was a significantly lower incidence of CTS procedures in the intervention group beyond four years of study enrollment (HR, 0.42; 95% CI, 0.22, 0.77; P = 0.006), which was significantly different from the HR in the first four years (p-value for interaction of 0.01, Table B in S1 File).

**Fig 3:**
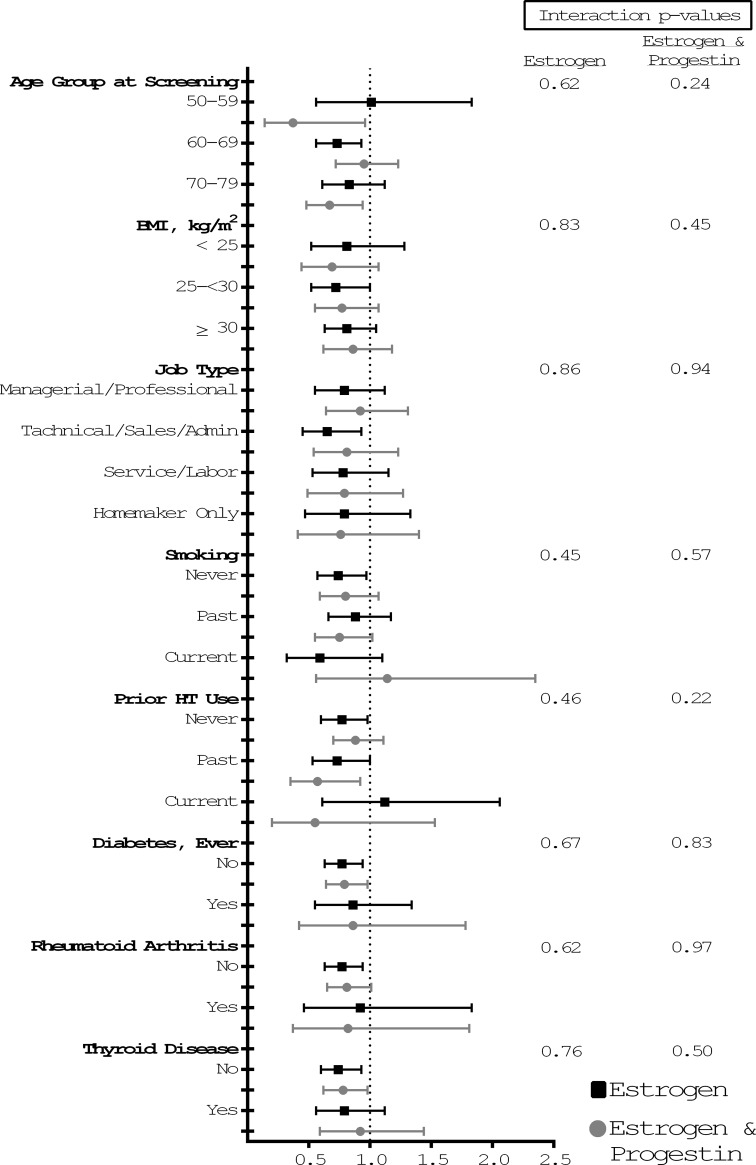
Subgroup analysis for development of carpal tunnel syndrome diagnosis by demographic and medical history. Subgroup analysis for development of CTS. Point estimates represent hazard ratios of the intervention compared to placebo and the whiskers are 95% confidence limits. BMI, Body Mass Index. There was no statistically significant interaction. The P values shown have not been adjusted for multiple comparisons.

No significant interactions for risk of CTS were found between study drug assignment and BMI, job type, smoking status, prior hormone use, history of diabetes, rheumatoid arthritis, and thyroid disease in the analyses (all P values for interaction ≥0.3) (Fig 3; Table B in S1 File). Sensitivity analyses accounting for non-adherence were performed and showed similar but slightly stronger protective effects of HT on the incidence of CTS compared to the primary analyses (Table C in S1 File).

## Discussion

After menopause, and in cases of iatrogenic premature menopause, estrogen is no longer synthesized by the ovaries and this has many side effects. Research on menopausal hormone therapy is important, as is the ongoing debate about benefits versus risks. Most recently, the US preventive Services Task Force presented a Recommendation Statement and Evidence Report that consider the risks and benefits of menopausal hormone therapy for primary prevention of chronic conditions.[25] In this report, it was clear that more research like what is presented in this paper is needed to be used by physicians to impart to their patients during discussions about therapies for menopausal symptoms like CTS.

These findings demonstrate, for the first time in secondary analysis of a randomized, double-blind trial of HT in postmenopausal women, that the risk of incident CTS was decreased by CEE or CEE+MPA, each compared to placebo. In both trials, use of HT reduced CTS risk by approximately 20%. Further, while not statistically significant in either trial, the risk of therapeutic CTS procedures was similarly decreased in both intervention groups. In analyses examining participant characteristics that may increase CTS risk, no effect modification was detected, supporting our hypothesis that HT may decrease the risk of CTS.

There are several pathophysiological hypotheses by which HT might influence the development of CTS. Changes in forearm fat content that occur during and after menopause, which may be mitigated or reversed by HT, could result in reduced risk of CTS.[15, 26] Also, estrogen and progesterone receptors are present in the transverse carpal ligament and flexor tenosynovium, suggesting that HT may have specific biologic effects by modulating pressure in the carpal tunnel.[27] Various estrogen preparations with or without progestin were found to have favorable effects on most soluble inflammatory markers including prostaglandin E2 and vascular endothelial factor (VEGF).[28, 29] These two markers are specifically excessively expressed in synovial biopsy tissue from patients with symptomatic CTS.[30]

Further, aromatase inhibitors, which may lower serum estrogen levels, can increase the incidence of CTS and arthralgia in breast cancer patients by contributing to tenosynovitis and edema specifically in the flexor compartments of the wrist.[31–34] The rapid drop in estrogen concentrations induced by aromatase inhibitors is believed to reduce the protective anti-nociceptive effect of estrogens, which decreases the threshold for pain stimuli, thereby exposing patients to symptoms from underlying musculoskeletal conditions such as CTS.[35] Additionally, menopause induced by surgery or radiation increases CTS incidence, an observation which supports our findings.[36, 37] Furthermore, the well-established positive immunomodulatory effect of HT in autoimmune and inflammatory conditions, such as multiple sclerosis and arthritis, suggest that withdrawal of endogenous estrogen facilitate the onset of inflammation, which may be offset by estrogen replacement.[38–40] It remains unclear, however, whether HT has the same protective effects that may reduce the incidence of CTS in postmenopausal women. Moreover, immunohistochemical studies suggest that estrogen receptors are upregulated in tenosynovial tissue in postmenopausal women with idiopathic CTS.[27, 41]

This is the first study to examine a potential causal relationship between HT and CTS in a large number of participants with several years of follow-up. Case reports[14] and observational studies in hemodialysis patients[42] suggested a protective role for HT. In the current study, CTS procedure rates were not statistically significantly different between the intervention and the placebo groups likely because of the number of procedures was relatively small decreasing the power to achieve statistical significance. However, the effect size was consistent with the CTS incidence. Interestingly, after 4 years of follow up in the CEE trial, CTS procedure rates were substantially higher in the placebo group compared to the intervention group. It is possible that in the first several years following a suspected CTS diagnosis, conservative management strategies were tried and might have provided short-term relief in some patients before surgery was advised, which may explain the noted temporal relationship.

Our findings may also suggest that CEE’s impact on CTS risk may differ from that of CEE+MPA. In the CEE+MPA trial, cumulative incidence curves diverged much earlier than in the CEE trial soon after the start of the trial. This finding may be due to different biologic effects of the interventions, possibly due to the progestin component in CEE+MPA that may have additional favorable effects. Another explanation may be attributed to differences in the study populations between the two trials. The latter hypothesis is supported by the different rates of CTS in the placebo arms between the two trials.[43]

The current study has several strengths such as its large study population, randomized clinical trial with intention-to-treat analysis which supports the generalizability of the results. It specifically investigated CTS as opposed to other studies in which general musculoskeletal diagnoses were the outcome of interest. Study limitations include the use of Medicare data for capturing CTS information; a method that has not been fully validated. However, Graham et al. reported that the correlation between CTS predicted by a regression model and a panel of physicians using ICD-9 codes was high (correlation coefficient was estimated at 0.71).[44] Procedure codes typically have a high positive predictive value for their indication, but this has not been formally validated for CTS procedures. Moreover, misclassification bias typically biases results toward the null, so is unlikely to explain our results. Since there was significant association between HT and CTS in the intervention portion of both trials, the results are unlikely to be explained by a significant misclassification of the outcomes. While women who had CTS at baseline or prior to randomization may not have been excluded because many WHI participants did not have Medicare claims prior to randomization and such information was not collected in the trial, the incidence in placebo and HT intervention groups is likely to have been balanced across randomization groups. This may suggest an effect of HT on treatment, rather than prevention of CTS. As in other randomized trials, women who volunteered to participate may have been healthier and more motivated to adhere to the study protocol, which may limit the generalizability of the findings to all postmenopausal women.

HT is no longer prescribed the way it was when the WHI was started fifteen years ago since there were abundant adverse outcomes associated with HT use. Therefore, this study is not encouraging HT use solely to prevent or treat CTS. However, current HT use are mostly related to quality of life and for some patients, especially those at increased risk of CTS, the evidence may inform clinical decisions depending on each patient’s predicted response or risk of disease. As we move more towards personalized medicine, it is important that we build the evidence in this area. Additionally, while these findings are interesting and novel, future research should explore the role of transdermal HT products for example on CTS incidence rates.

## Conclusions

To our knowledge, this is the first large-scale observational analysis in a randomized trial population where CTS risk, a common and chronic problem that affects functioning and productivity, is suggested to be decreased by hormonal intervention. CTS could be added to the list of outcomes that may inform the decision-making process for women considering menopausal HT, particularly those who have other risk factors for CTS. The preventive effect of both CEE and CEE+MPA may provide an opportunity to explore a potential therapeutic role of other types of HT such as transdermal patches for patients with prevalent CTS. Future clinical trials with CTS as a predefined endpoint are needed to examine and quantify this relationship.

## Supporting information

S1 File **Table A. Subgroup Analysis of the Number, Annualized Incidence, and Hazard of Carpal Tunnel Syndrome Diagnosis in Both Women’s Health Initiative (WHI) Trials.** ^1^The Cox proportional hazards regression model stratified by randomization age strata (50–54, 55–59, 60–69, 70–79) and Dietary Modification Trial randomization arm.^2^ Tests for interaction with randomization arm based on product terms between arm and linear age, linear trend for BMI (coded 1–3), and categorical factors for job type, smoking, prior HT use, diabetes, rheumat**oid arthritis and thyroid disease in Cox proportional hazards regression models stratified as above. Table B. Incidence of Carpal Tunnel Syndrome (CTS) Diagnosis during the Conjugated Equine Estrogens (CEE) Trial Intervention Period by years of follow-up (N = 6833). Sample in Center for Medicare & Medicaid Services (CMS) at randomization or later aged in during intervention.** ^1^ The Cox proportional hazards regression model stratified by randomization age strata (50–54, 55–59, 60–69, 70–79) and Dietary Modification Trial randomization arm. ^2^Test for difference of hazard ratios between ≤ vs. > 3 years p-value = 0.0126. 3Test for difference of hazard ratios between ≤ vs. > 4 years p-value = 0.0108. **Table C. Incidence of Carpal Tunnel Syndrome (CTS) Diagnosis During and After Hormone Trial Intervention Periods Adjusted for Drug Non-adherence: sample in Center for Medicare & Medicaid Services CMS at randomization or later aged in during intervention.** Follow-up time, mean (SD) for CEE trial = 3.3 (2.6) years and for CEE+MPA trial = 2.9 (2.1) years. Sensitivity analyses: Cox PH models adjusted for non-adherence (follow-up time censored 6 months after becoming non-adherent and weighted by the inverse of the participant’s probability of adherence). Participants who became non-adherent before the start of their CMS coverage are excluded (n = 2,716). 1 The Cox proportional hazards regression model stratified by randomization age strata (50–54, 55–59, 60–69, 70–79) and Dietary Modification Trial randomization arm.
(DOCX)

## References

[1] Thomsen NO, Björk J, Cederlund RI. Health-related quality of life 5 years after carpal tunnel release among patients with diabetes: a prospective study with matched controls. BMC endocrine disorders. 2014;14(1):85. 25326166 10.1186/1472-6823-14-85 PMC4203934

[2] Sperka P, Cherry N, Burnham R, Beach J. Impact of compensation on work outcome of carpal tunnel syndrome. Occupational medicine (Oxford, England). 2008;58(7):490–5. Epub 2008/08/23. 10.1093/occmed/kqn099 . 18718899 10.1093/occmed/kqn099

[3] Foley M, Silverstein B, Polissar N. The economic burden of carpal tunnel syndrome: Long-term earnings of CTS claimants in Washington State. American journal of industrial medicine. 2007;50(3):155–72. Epub 2007/01/12. 10.1002/ajim.20430 . 17216630 10.1002/ajim.20430

[4] Chatterjee A, McCarthy JE, Montagne SA, Leong K, Kerrigan CL. A cost, profit, and efficiency analysis of performing carpal tunnel surgery in the operating room versus the clinic setting in the United States. Ann Plast Surg. 2011;66(3):245–8. 10.1097/SAP.0b013e3181db7784 . 21042185 10.1097/SAP.0b013e3181db7784

[5] Jerosch-Herold C, Shepstone L, Wilson EC, Dyer T, Blake J. Clinical course, costs and predictive factors for response to treatment in carpal tunnel syndrome: the PALMS study protocol. BMC musculoskeletal disorders. 2014;15:35 Epub 2014/02/11. 10.1186/1471-2474-15-35 ; PubMed Central PMCID: PMCPMC3921988. 24507749 10.1186/1471-2474-15-35 PMC3921988

[6] Dale AM, Harris-Adamson C, Rempel D, Gerr F, Hegmann K, Silverstein B, et al Prevalence and incidence of carpal tunnel syndrome in US working populations: pooled analysis of six prospective studies. Scandinavian journal of work, environment & health. 2013;39(5):495–505. Epub 2013/02/21. 10.5271/sjweh.3351 ; PubMed Central PMCID: PMCPMC4042862. 23423472 10.5271/sjweh.3351 PMC4042862

[7] Atroshi I, Gummesson C, Johnsson R, Ornstein E, Ranstam J, Rosen I. Prevalence of carpal tunnel syndrome in a general population. Jama. 1999;282(2):153–8. Epub 1999/07/20. . 10411196 10.1001/jama.282.2.153

[8] Stevens JC, Sun S, Beard CM, O'Fallon WM, Kurland LT. Carpal tunnel syndrome in Rochester, Minnesota, 1961 to 1980. Neurology. 1988;38(1):134–8. Epub 1988/01/01. . 3336444 10.1212/wnl.38.1.134

[9] Mondelli M, Giannini F, Giacchi M. Carpal tunnel syndrome incidence in a general population. Neurology. 2002;58(2):289–94. Epub 2002/01/24. . 11805259 10.1212/wnl.58.2.289

[10] Stocks SJ, McNamee R, van der Molen HF, Paris C, Urban P, Campo G, et al Trends in incidence of occupational asthma, contact dermatitis, noise-induced hearing loss, carpal tunnel syndrome and upper limb musculoskeletal disorders in European countries from 2000 to 2012. Occupational and environmental medicine. 2015;72(4):294–303. Epub 2015/01/13. 10.1136/oemed-2014-102534 . 25575531 10.1136/oemed-2014-102534

[11] Kaplan Y, Kurt SG, Karaer H. Carpal tunnel syndrome in postmenopausal women. Journal of the neurological sciences. 2008;270(1–2):77–81. Epub 2008/03/08. 10.1016/j.jns.2008.02.003 . 18325536 10.1016/j.jns.2008.02.003

[12] Oktayoglu P, Nas K, Kilinc F, Tasdemir N, Bozkurt M, Yildiz I. Assessment of the Presence of Carpal Tunnel Syndrome in Patients with Diabetes Mellitus, Hypothyroidism and Acromegaly. Journal of clinical and diagnostic research: JCDR. 2015;9(6):OC14–8. Epub 2015/08/13. 10.7860/JCDR/2015/13149.6101 ; PubMed Central PMCID: PMCPMC4525537. 26266148 10.7860/JCDR/2015/13149.6101 PMC4525537

[13] Niver GE, Ilyas AM. Carpal tunnel syndrome after distal radius fracture. The Orthopedic clinics of North America. 2012;43(4):521–7. Epub 2012/10/03. 10.1016/j.ocl.2012.07.021 . 23026468 10.1016/j.ocl.2012.07.021

[14] Confino-Cohen R, Lishner M, Savin H, Lang R, Ravid M. Response of carpal tunnel syndrome to hormone replacement therapy. Bmj. 1991;303(6816):1514 ; PubMed Central PMCID: PMCPMC1671863. 1838291 10.1136/bmj.303.6816.1514 PMC1671863

[15] Hall GM, Spector TD, Studd JW. Carpal tunnel syndrome and hormone replacement therapy. Bmj. 1992;304(6823):382 Epub 1992/02/08. ; PubMed Central PMCID: PMCPMC1881252. 1540746 10.1136/bmj.304.6823.382 PMC1881252

[16] Geoghegan JM, Clark DI, Bainbridge LC, Smith C, Hubbard R. Risk factors in carpal tunnel syndrome. Journal of hand surgery (Edinburgh, Scotland). 2004;29(4):315–20. Epub 2004/07/06. 10.1016/j.jhsb.2004.02.009 . 15234492 10.1016/j.jhsb.2004.02.009

[17] Ferry S, Hannaford P, Warskyj M, Lewis M, Croft P. Carpal tunnel syndrome: a nested case-control study of risk factors in women. American journal of epidemiology. 2000;151(6):566–74. Epub 2000/03/25. . 10733038 10.1093/oxfordjournals.aje.a010244

[18] Jansson C, Johansson S, Lindh-Astrand L, Hoffmann M, Hammar M. The prevalence of symptoms possibly related to the climacteric in pre- and postmenopausal women in Linkoping, Sweden. Maturitas. 2003;45(2):129–35. . 12787971 10.1016/s0378-5122(03)00127-0

[19] Rossouw JE, Anderson GL, Prentice RL, LaCroix AZ, Kooperberg C, Stefanick ML, et al Risks and benefits of estrogen plus progestin in healthy postmenopausal women: principal results From the Women's Health Initiative randomized controlled trial. Jama. 2002;288(3):321–33. Epub 2002/07/19. . 12117397 10.1001/jama.288.3.321

[20] Design of the Women's Health Initiative clinical trial and observational study. The Women's Health Initiative Study Group. Controlled clinical trials. 1998;19(1):61–109. Epub 1998/03/11. . 9492970 10.1016/s0197-2456(97)00078-0

[21] Anderson GL, Limacher M, Assaf AR, Bassford T, Beresford SA, Black H, et al Effects of conjugated equine estrogen in postmenopausal women with hysterectomy: the Women's Health Initiative randomized controlled trial. Jama. 2004;291(14):1701–12. Epub 2004/04/15. 10.1001/jama.291.14.1701 . 15082697 10.1001/jama.291.14.1701

[22] Kalbfleisch JD, Prentice RL. The statistical analysis of failure time data: John Wiley & Sons; 2011.

[23] Robins JM, Finkelstein DM. Correcting for noncompliance and dependent censoring in an AIDS clinical trial with inverse probability of censoring weighted (IPCW) log‐rank tests. Biometrics. 2000;56(3):779–88. 10985216 10.1111/j.0006-341x.2000.00779.x

[24] Stefanick ML, Cochrane BB, Hsia J, Barad DH, Liu JH, Johnson SR. The Women's Health Initiative postmenopausal hormone trials: overview and baseline characteristics of participants. Annals of epidemiology. 2003;13(9 Suppl):S78–86. Epub 2003/10/25. . 14575940 10.1016/s1047-2797(03)00045-0

[25] Lewis CE, Wellons MF. Menopausal hormone therapy for primary prevention of chronic disease. JAMA. 2017;318(22):2187–9. 10.1001/jama.2017.16974 29234792 10.1001/jama.2017.16974

[26] Gambacciani M, Ciaponi M, Cappagli B, De Simone L, Orlandi R, Genazzani AR. Prospective evaluation of body weight and body fat distribution in early postmenopausal women with and without hormonal replacement therapy. Maturitas. 2001;39(2):125–32. Epub 2001/08/22. . 11514111 10.1016/s0378-5122(01)00194-3

[27] Toesca A, Pagnotta A, Zumbo A, Sadun R. Estrogen and progesterone receptors in carpal tunnel syndrome. Cell biology international. 2008;32(1):75–9. Epub 2007/10/24. 10.1016/j.cellbi.2007.08.014 . 17951080 10.1016/j.cellbi.2007.08.014

[28] Georgiadou P, Sbarouni E. Effect of hormone replacement therapy on inflammatory biomarkers. Advances in clinical chemistry. 2009;47:59–93. Epub 2009/07/29. . 19634777 10.1016/s0065-2423(09)47003-3

[29] Sumino H, Nakamura T, Ichikawa S, Kanda T, Sakamaki T, Sato K, et al Serum level of vascular endothelial growth factor is decreased by hormone replacement therapy in postmenopausal women without hypercholesterolemia. Atherosclerosis. 2000;148(1):189–95. Epub 1999/12/02. . 10580185 10.1016/s0021-9150(99)00262-2

[30] Hirata H, Nagakura T, Tsujii M, Morita A, Fujisawa K, Uchida A. The relationship of VEGF and PGE2 expression to extracellular matrix remodelling of the tenosynovium in the carpal tunnel syndrome. The Journal of pathology. 2004;204(5):605–12. Epub 2004/11/13. 10.1002/path.1673 . 15538733 10.1002/path.1673

[31] Din OS, Dodwell D, Wakefield RJ, Coleman RE. Aromatase inhibitor-induced arthralgia in early breast cancer: what do we know and how can we find out more? Breast cancer research and treatment. 2010;120(3):525–38. Epub 2010/02/17. 10.1007/s10549-010-0757-7 . 20157776 10.1007/s10549-010-0757-7

[32] Sestak I, Sapunar F, Cuzick J. Aromatase inhibitor-induced carpal tunnel syndrome: results from the ATAC trial. Journal of clinical oncology: official journal of the American Society of Clinical Oncology. 2009;27(30):4961–5. Epub 2009/09/16. 10.1200/JCO.2009.22.0236 . 19752338 10.1200/JCO.2009.22.0236

[33] Riley G. The pathogenesis of tendinopathy. A molecular perspective. Rheumatology (Oxford, England). 2004;43(2):131–42. Epub 2003/07/18. 10.1093/rheumatology/keg448 . 12867575 10.1093/rheumatology/keg448

[34] Morales L, Pans S, Verschueren K, Van Calster B, Paridaens R, Westhovens R, et al Prospective study to assess short-term intra-articular and tenosynovial changes in the aromatase inhibitor-associated arthralgia syndrome. Journal of clinical oncology: official journal of the American Society of Clinical Oncology. 2008;26(19):3147–52. 10.1200/JCO.2007.15.4005 . 18474874 10.1200/JCO.2007.15.4005

[35] Bliss JM, Kilburn LS, Coleman RE, Forbes JF, Coates AS, Jones SE, et al Disease-related outcomes with long-term follow-up: an updated analysis of the intergroup exemestane study. Journal of clinical oncology: official journal of the American Society of Clinical Oncology. 2012;30(7):709–17. Epub 2011/11/02. 10.1200/JCO.2010.33.7899 . 22042946 10.1200/JCO.2010.33.7899

[36] Bjorkqvist SE, Lang AH, Punnonen R, Rauramo L. Carpal Tunnel syndrome in ovariectomized women. Acta obstetricia et gynecologica Scandinavica. 1977;56(2):127–30. Epub 1977/01/01. . 855654 10.3109/00016347709158354

[37] Pascual E, Giner V, Arostegui A, Conill J, Ruiz MT, Pico A. Higher incidence of carpal tunnel syndrome in oophorectomized women. British journal of rheumatology. 1991;30(1):60–2. Epub 1991/02/01. . 1991220 10.1093/rheumatology/30.1.60

[38] Vegeto E, Ciana P, Maggi A. Estrogen and inflammation: hormone generous action spreads to the brain. Molecular psychiatry. 2002;7(3):236–8. Epub 2002/03/29. 10.1038/sj.mp.4001007 . 11920150 10.1038/sj.mp.4001007

[39] Ali ES, Mangold C, Peiris AN. Estriol: emerging clinical benefits. Menopause (New York, NY). 2017 Epub 2017/04/05. 10.1097/GME.0000000000000855 . 28375935 10.1097/GME.0000000000000855

[40] Straub RH. The complex role of estrogens in inflammation. Endocr Rev. 2007;28(5):521–74. 10.1210/er.2007-0001 . 17640948 10.1210/er.2007-0001

[41] Kim JK, Hann HJ, Kim MJ, Kim JS. The expression of estrogen receptors in the tenosynovium of postmenopausal women with idiopathic carpal tunnel syndrome. Journal of orthopaedic research: official publication of the Orthopaedic Research Society. 2010;28(11):1469–74. Epub 2010/09/28. 10.1002/jor.21160 . 20872583 10.1002/jor.21160

[42] Hamano T, Fujii N, Ito T, Imai E, Mikami S, Katayama M, et al [Low dose estrogen replacement therapy (ERT) for postmenopausal hemodialysis (HD) patients]. Clinical calcium. 2005;15 Suppl 1:161–6; discussion 6. Epub 2005/11/11. . 16279020

[43] Manson JE, Chlebowski RT, Stefanick ML, Aragaki AK, Rossouw JE, Prentice RL, et al Menopausal hormone therapy and health outcomes during the intervention and extended poststopping phases of the Women's Health Initiative randomized trials. Jama. 2013;310(13):1353–68. Epub 2013/10/03. 10.1001/jama.2013.278040 ; PubMed Central PMCID: PMCPMC3963523. 24084921 10.1001/jama.2013.278040 PMC3963523

[44] Graham B, Regehr G, Naglie G, Wright JG. Development and validation of diagnostic criteria for carpal tunnel syndrome. The Journal of hand surgery. 2006;31(6):919–24. Epub 2006/08/05. . 16886290

