# Supplementary material for: Menopausal hormone therapy and the incidence of carpal tunnel syndrome in postmenopausal women: Findings from the Women’s Health Initiative
Source: PLoS One. 2018 Dec 4;13(12):e0207509. doi: 10.1371/journal.pone.0207509 (PMC6279038; doi:10.1371/journal.pone.0207509)
Supplement: S1 File — Table A. Subgroup Analysis of the Number, Annualized Incidence, and Hazard of Carpal Tunnel Syndrome Diagnosis in Both Women’s Health Initiative (WHI) Trials. 1The Cox proportional hazards regression model stratified by randomization age strata (50–54, 55–59, 60–69, 70–79) and Dietary Modification Trial randomization arm.2 Tests for interaction with randomization arm based on product terms between arm and linear age, linear trend for BMI (coded 1–3), and categorical factors for job type, smoking, prior HT use, diabetes, rheumatoid arthritis and thyroid disease in Cox proportional hazards regression models stratified as above. Table B. Incidence of Carpal Tunnel Syndrome (CTS) Diagnosis during the Conjugated Equine Estrogens (CEE) Trial Intervention Period by years of follow-up (N = 6833). Sample in Center for Medicare & Medicaid Services (CMS) at randomization or later aged in during intervention. 1 The Cox proportional hazards regression model stratified by randomization age strata (50–54, 55–59, 60–69, 70–79) and Dietary Modification Trial randomization arm. 2Test for difference of hazard ratios between ≤ vs. > 3 years p-value = 0.0126. 3Test for difference of hazard ratios between ≤ vs. > 4 years p-value = 0.0108. Table C. Incidence of Carpal Tunnel Syndrome (CTS) Diagnosis During and After Hormone Trial Intervention Periods Adjusted for Drug Non-adherence: sample in Center for Medicare & Medicaid Services CMS at randomization or later aged in during intervention. Follow-up time, mean (SD) for CEE trial = 3.3 (2.6) years and for CEE+MPA trial = 2.9 (2.1) years. Sensitivity analyses: Cox PH models adjusted for non-adherence (follow-up time censored 6 months after becoming non-adherent and weighted by the inverse of the participant’s probability of adherence). Participants who became non-adherent before the start of their CMS coverage are excluded (n = 2,716). 1 The Cox proportional hazards regression model stratified by randomization age strata (50–54, 55–59, 60–6 [file pone.0207509.s001.docx]

| **Table A: Subgroup Analysis of the Number, Annualized Incidence, and Hazard of Carpal Tunnel Syndrome Diagnosis in Both Women’s Health Initiative (WHI) Trials** | | | | | | | | |
| --- | --- | --- | --- | --- | --- | --- | --- | --- |
|  | **Conjugated Equine Estrogens Trial** | | |  | **Estrogen & Progestin Trial** | | |  |
|  | **CEE,**  **N/Ann. %** | **Placebo,**  **N/Ann. %** | **HR^1^**  **(95% CI)** | **Interaction**  **p-value^2^** | **CEE+MPA,**  **N/Ann. %** | **Placebo,**  **N/Ann. %** | **HR^1^**  **(95% CI)** | **Interaction**  **p-value^2^** |
| **Age group at screening** |  |  |  | 0.616 |  |  |  | 0.236 |
| 50-59 | 22/2.01 | 22/2.02 | 1.01 (0.56-1.83) |  | 6/0.90 | 14/2.45 | 0.37 (0.14-0.96) |  |
| 60-69 | 104/1.24 | 146/1.71 | 0.73 (0.56-0.93) |  | 109/1.03 | 108/1.08 | 0.95 (0.72-1.23) |  |
| 70-79 | 77/1.49 | 94/1.80 | 0.83 (0.61-1.12) |  | 58/0.99 | 81/1.46 | 0.67 (0.48-0.94) |  |
| **BMI, kg/m^2^** |  |  |  | 0.832 |  |  |  | 0.448 |
| < 25 | 34/1.04 | 42/1.27 | 0.81 (0.52-1.28) |  | 34/0.65 | 47/0.92 | 0.69 (0.44-1.07) |  |
| 25-< 30 | 60/1.17 | 87/1.61 | 0.72 (0.52-1.00) |  | 64/1.04 | 79/1.34 | 0.77 (0.55-1.07) |  |
| ≥ 30 | 109/1.77 | 132/2.17 | 0.81 (0.63-1.05) |  | 73/1.29 | 76/1.53 | 0.86 (0.62-1.18) |  |
| **Job type** |  |  |  | 0.859 |  |  |  | 0.937 |
| Managerial/Professional | 53/1.33 | 71/1.72 | 0.79 (0.55-1.12) |  | 58/1.12 | 65/1.23 | 0.92 (0.64-1.31) |  |
| Technical/Sales/Admin | 47/1.22 | 73/1.86 | 0.65 (0.45-0.93) |  | 42/1.00 | 50/1.19 | 0.81 (0.54-1.23) |  |
| Service/Labor | 46/1.51 | 57/1.93 | 0.78 (0.53-1.15) |  | 31/1.06 | 39/1.36 | 0.79 (0.49-1.27) |  |
| Homemaker only | 26/1.68 | 30/2.06 | 0.79 (0.47-1.33) |  | 18/1.02 | 25/1.37 | 0.76 (0.41-1.40) |  |
| **Smoking** |  |  |  | 0.453 |  |  |  | 0.574 |
| Never | 95/1.23 | 128/1.64 | 0.74 (0.57-0.97) |  | 83/0.95 | 96/1.17 | 0.80 (0.59-1.07) |  |
| Past | 89/1.61 | 103/1.83 | 0.88 (0.66-1.17) |  | 73/1.05 | 90/1.41 | 0.75 (0.55-1.02) |  |
| Current | 16/1.28 | 27/2.15 | 0.59 (0.32-1.10) |  | 16/1.23 | 14/1.15 | 1.14 (0.56-2.35) |  |
| **Prior HT use** |  |  |  | 0.458 |  |  |  | 0.219 |
| Never | 115/1.47 | 150/1.91 | 0.77 (0.60-0.98) |  | 140/1.06 | 148/1.20 | 0.88 (0.70-1.11) |  |
| Past | 66/1.20 | 92/1.63 | 0.73 (0.53-1.00) |  | 27/0.82 | 45/1.43 | 0.57 (0.35-0.92) |  |
| Current | 22/1.68 | 20/1.45 | 1.12 (0.61-2.06) |  | 6/1.03 | 10/1.90 | 0.55 (0.20-1.53) |  |
| **Diabetes, ever** |  |  |  | 0.673 |  |  |  | 0.830 |
| No | 168/1.27 | 219/1.63 | 0.77 (0.63-0.94) |  | 160/0.99 | 186/1.23 | 0.79 (0.64-0.98) |  |
| Yes | 35/2.53 | 43/2.96 | 0.86 (0.55-1.34) |  | 13/1.36 | 17/1.73 | 0.86 (0.42-1.78) |  |
| **Rheumatoid arthritis** |  |  |  | 0.624 |  |  |  | 0.969 |
| No | 170/1.35 | 226/1.76 | 0.77 (0.63-0.94) |  | 144/0.96 | 173/1.19 | 0.81 (0.65-1.01) |  |
| Yes | 17/1.74 | 16/1.85 | 0.92 (0.46-1.83) |  | 12/1.56 | 13/1.93 | 0.82 (0.37-1.81) |  |
| **Thyroid disease** |  |  |  | 0.762 |  |  |  | 0.498 |
| No | 135/1.26 | 193/1.70 | 0.74 (0.60-0.93) |  | 133/1.01 | 161/1.30 | 0.78 (0.62-0.98) |  |
| Yes | 60/1.59 | 66/1.97 | 0.79 (0.56-1.12) |  | 40/1.04 | 39/1.10 | 0.92 (0.59-1.44) |  |
|  | | | | | | | | |

^1^The Cox proportional hazards regression model stratified by randomization age strata (50-54, 55-59, 60-69, 70-79) and Dietary Modification Trial randomization arm.

^2^ Tests for interaction with randomization arm based on product terms between arm and linear age, linear trend for BMI (coded 1-3), and categorical factors for job type, smoking, prior HT use, diabetes, rheumatoid arthritis and thyroid disease in Cox proportional hazards regression models stratified as above.

**Table B. Incidence of Carpal Tunnel Syndrome (CTS) Diagnosis during the Conjugated Equine Estrogens (CEE) Trial Intervention Period by years of follow-up (N=6833). Sample in Center for Medicare & Medicaid Services (CMS) at randomization or later aged in during intervention.**

|  | | | | |
| --- | --- | --- | --- | --- |
|  | CEE (N=3,380) | Placebo (N=3,453) | HR^1^ (95% CI) | p-value^1^ |
|  | No. of events | No. of events |  |  |
| **CTS diagnosis^2^** |  |  |  |  |
| Overall | 203 | 262 | 0.78 (0.65-0.94) | 0.009 |
| ≤ 3 years | 121 | 127 | 0.97 (0.76-1.25) | 0.831 |
| > 3 years | 82 | 135 | 0.61 (0.46-0.80) | 0.001 |
| **CTS procedure^3^** |  |  |  |  |
| Overall | 52 | 69 | 0.77 (0.54-1.11) | 0.164 |
| ≤ 4 years | 38 | 34 | 1.14 (0.72-1.81) | 0.583 |
| > 4 years | 14 | 35 | 0.42 (0.22-0.77) | 0.006 |
| ^1^ The Cox proportional hazards regression model stratified by randomization age strata (50-54, 55-59, 60-69, 70-79) and Dietary Modification Trial randomization arm. ^2^Test for difference of hazard ratios between ≤ vs. > 3 years p-value=0.0126.  ^3^Test for difference of hazard ratios between ≤ vs. > 4 years p-value=0.0108. | | | | |

**Table C. Incidence of Carpal Tunnel Syndrome (CTS) Diagnosis During and After Hormone Trial Intervention Periods Adjusted for Drug Non-adherence: sample in Center for Medicare & Medicaid Services CMS at randomization or later aged in during intervention**

|  | | | | | | |
| --- | --- | --- | --- | --- | --- | --- |
|  | **Conjugated Equine Estrogens Trial** | | | **Estrogen & Progestin Trial** | | |
|  | CEE | Placebo | p-value^1^ | CEE+MPA | Placebo | p-value^1^ |
| **CTS diagnosis** | N=2,725 | N=2,783 |  | N=3,909 | N=3,920 |  |
| HR^1^ (95% CI) | 0.68 (0.56-0.89) | Ref | 0.0059 | 0.74 (0.56-0.78) | Ref | 0.0356 |
| Events, N (%) | 103 (3.8) | 149 (5.4) |  | 91 (2.3) | 136 (3.5) |  |
| Annualized incidence (%) | 1.23 | 1.70 |  | 0.89 | 1.22 |  |
| **CTS procedure** | N=2,725 | N=2,783 |  | N=3,909 | N=3,920 |  |
| HR^1^ (95% CI) | 0.61 (0.37-1.00) | Ref | 0.0519 | 0.64 (0.35-1.15) | Ref | 0.1371 |
| Events, N (%) | 28 (1.0) | 39 (1.4) |  | 19 (0.5) | 35 (0.9) |  |
| Annualized incidence (%) | 0.33 | 0.43 |  | 0.18 | 0.31 |  |
|  | | | | | | |

Follow-up time, mean (SD) for CEE trial = 3.3 (2.6) years and for CEE+MPA trial = 2.9 (2.1) years.

Sensitivity analyses: Cox PH models adjusted for non-adherence (follow-up time censored 6 months after becoming non-adherent and weighted by the inverse of the participant’s probability of adherence). Participants who became non-adherent before the start of their CMS coverage are excluded (n=2,716).

^1^ The Cox proportional hazards regression model stratified by randomization age strata (50-54, 55-59, 60-69, 70-79) and Dietary Modification Trial randomization arm.
